# Influence of Surface Modification and Artificial Aging on *Streptococcus mutans* Biofilm Formation and Oral Cell Responses to Orthodontic PMMA: An In Vitro Study

**DOI:** 10.3390/ma19214474

**Published:** 2026-10-23

**Authors:** Lucia Giannini, Marco Farronato, Antonino Manti, Giovanni Cattaneo, Gianna Dipalma, Cinzia Maspero

**Affiliations:** 1 Fondazione IRCCS Ca’ Granda Ospedale Maggiore Policlinico, 20122 Milan, Italy; dott.lucia.giannini@gmail.com (L.G.);; 2 Department of Biomedical, Surgical and Dental Sciences, University of Milan, 20122 Milan, Italy; antonino.manti@unimi.it (A.M.);; 3 Department of Interdisciplinary Medicine, University of Bari Aldo Moro, 70121 Bari, Italy; 4 Department of Life Science, Health and Health Professions, Link Campus University, 00165 Rome, Italy

**Keywords:** polymethyl methacrylate, orthodontic acrylic, 2-methacryloyloxyethyl phosphorylcholine, MPC, PMBPAz, zwitterionic coating, *Streptococcus mutans*, biofilm, artificial aging, gingival fibroblasts

## Abstract

Background/Objectives: Photoreactive zwitterionic PMBPAz coatings have previously been used to modify dental PMMA, but their integrated performance on clinically polished orthodontic PMMA under a specified composite-aging challenge remains insufficiently characterized. This in vitro study compared untreated, polished, and PMBPAz-MPC-coated PMMA before and after laboratory aging. Materials and Methods: Heat-polymerized PMMA specimens were tested for early *Streptococcus mutans* adhesion, 24 h biofilm indices, surface chemistry and wettability, protein adsorption, mechanical properties, and human gingival fibroblast responses. A separate four-time-point MPC-PMMA series evaluated artificial saliva immersion alone, while the composite-aged groups also underwent thermocycling and simulated toothbrushing. Results: Compared with polished PMMA, MPC-PMMA showed lower 2 h adhesion by 0.73 log10 CFU/cm^2^ (81%) before aging and 0.62 log10 CFU/cm^2^ (76%) after composite aging; the corresponding decreases in crystal violet biomass were 44.6% and 37.9%. In the saliva-only series, coating-associated XPS phosphorus decreased from 3.8 to 3.3 at.%, and the contact angle increased from 18.6° to 21.7° at T28; following composite aging, the corresponding values were 2.9 at.% and 24.3°. Extract-exposed fibroblasts showed group-dependent changes in LDH and inflammatory mediators. Conclusions: Under the specified in vitro conditions, PMBPAz-MPC treatment of polished orthodontic PMMA was associated with reduced early *S. mutans* colonization, including after composite aging. The findings do not establish clinical efficacy or long-term biological safety.

## 1. Introduction

Polymethyl methacrylate (PMMA) remains widely used for removable orthodontic appliances, functional appliances, retainers, and acrylic components because of its ease of processing, low weight, dimensional stability, and clinical versatility. However, polymeric surfaces can provide retentive niches for microbial accumulation, particularly when surface irregularities, porosity, or hydrophobic interactions favor early bacterial attachment. In our previous standardized comparison of orthodontic materials, PMMA showed the highest *S. mutans* early adhesion, biofilm biomass, and metabolic activity, while surface roughness and contact angle were positively associated with bacterial colonization [1]. This finding provided the experimental basis for a second-stage study focused on targeted surface engineering rather than another descriptive comparison among unrelated substrates.

An effective anti-biofilm strategy for orthodontic PMMA should ideally interfere with the conditioning events that precede microbial attachment rather than depend exclusively on continuous release of antibacterial agents. Zwitterionic 2-methacryloyloxyethyl phosphorylcholine (MPC) polymers form highly hydrated interfaces with strong resistance to nonspecific protein adsorption. Photoreactive MPC copolymers containing n-butyl methacrylate (BMA) and 2-methacryloyloxyethyl-4-azidobenzoate (MPAz), collectively referred to as PMBPAz, can be covalently anchored to polymeric substrates by ultraviolet activation of the azide groups [2,3]. On PMMA dental resin, PMBPAz has inhibited *S. mutans* plaque accumulation, while clinical denture studies have reported lower plaque deposition after MPC-polymer treatment [4,5]. Anti-biofilm modification of PMMA has also been investigated for orthodontic applications using other surface or nanomaterial strategies [6].

The novelty of the present work is not the synthesis of PMBPAz, its application to PMMA, or the general observation of reduced plaque deposition, all of which have precedents [2,3,4,5]. Rather, the study addresses a narrower materials science question: whether this established photoreactive coating adds anti-adhesive activity beyond standardized clinical polishing on a single orthodontic PMMA substrate, and whether a coordinated set of bacterial, physicochemical, mechanical, and extract-based cellular outcomes remains favorable after a combined immersion–thermal–brushing challenge. This integrated experimental comparison should not be interpreted as a claim of first use of MPC on acrylic dental materials.

Freshly prepared surfaces may not represent the condition of an orthodontic appliance after repeated exposure to saliva, temperature changes, and mechanical cleaning. Hydrolytic exposure, thermocycling, and toothbrushing can modify the surface and mechanical behavior of dental polymers [7,8]. For a surface-bound anti-fouling treatment, durability is therefore part of efficacy: the coating should retain its chemical signature and wetting behavior, and its microbiological effect should remain detectable after simulated use.

Biological safety is equally important. A surface treatment that reduces bacterial adhesion would have limited translational value if it adversely affected oral soft-tissue cells or compromised the underlying PMMA. PMMA-based dental materials can differ in gingival fibroblast responses according to formulation and processing [9]. Accordingly, biological validation of a modified orthodontic PMMA should integrate microbiological outcomes with cytocompatibility, inflammatory markers, and mechanical performance.

Removable functional appliances incorporating acrylic components are widely used in interceptive orthodontics [10,11,12,13]. This clinical use provides a rationale for evaluating whether surface modification of PMMA can reduce bacterial colonization while preserving cytocompatibility and mechanical performance.

The aim of this in vitro study was to evaluate a previously described photoreactive PMBPAz-MPC coating when applied to clinically polished orthodontic PMMA. The primary objective was the incremental reduction in 2 h *S. mutans* adhesion relative to polished, uncoated PMMA. Secondary objectives were 24 h biofilm biomass and metabolic activity, coating-associated surface properties and their persistence during hydrolytic exposure and after composite artificial aging, protein adsorption, material mechanics, and responses of gingival fibroblasts to material extracts.

The null hypotheses were that: (1) surface condition (UT-PMMA, P-PMMA, or MPC-PMMA) would not influence *S. mutans* adhesion or biofilm formation; (2) simulated intraoral aging would not modify these outcomes; (3) there would be no surface condition × aging interaction; (4) the MPC-containing coating would not alter protein adsorption or surface physicochemical characteristics; and (5) the coating would not influence mechanical properties, cytocompatibility, or inflammatory mediator release.

## 2. Materials and Methods

### 2.1. Study Design and Experimental Groups

This in vitro study used a 3 × 2 factorial design with surface (untreated, clinically polished, or MPC-coated) and aging conditions (fresh or aged) as the two fixed experimental factors. All principal groups were fabricated from the same heat-polymerized PMMA substrate, with standardized specimen geometry across groups. This design isolated the effect of the surface intervention from differences in bulk material composition or specimen geometry.

- UT-PMMA: untreated PMMA prepared using the standardized fabrication protocol and receiving no final clinical polishing or MPC coating;
- P-PMMA: PMMA subjected to the standardized clinical polishing protocol, serving as the clinically relevant surface control;
- MPC-PMMA: PMMA subjected to the identical polishing protocol followed by a photoreactive MPC-containing poly(MPC-co-BMA-co-MPAz) (PMBPAz) coating.

Each surface condition was evaluated in both fresh and aged states, producing six primary experimental conditions (UT-F, UT-A, P-F, P-A, MPC-F, and MPC-A). Stainless steel was not included in the primary design because the objective was to test a targeted surface treatment of a single PMMA substrate. The previously published low-colonization stainless-steel group was retained only as historical biological context [1].

No matched sham-treated polished control (vehicle application plus UV exposure without PMBPAz) was included. Therefore, P-PMMA provides the clinically relevant uncoated comparator but cannot fully separate the contribution of PMBPAz chemistry from all processing-associated effects (vehicle exposure, irradiation, or subsequent rinsing). An unirradiated polymer-coated process control, which evaluates the role of UV activation in coating retention, would not replace the missing solvent/UV/rinse-only sham comparator (Table 1).

**Figure 1:**
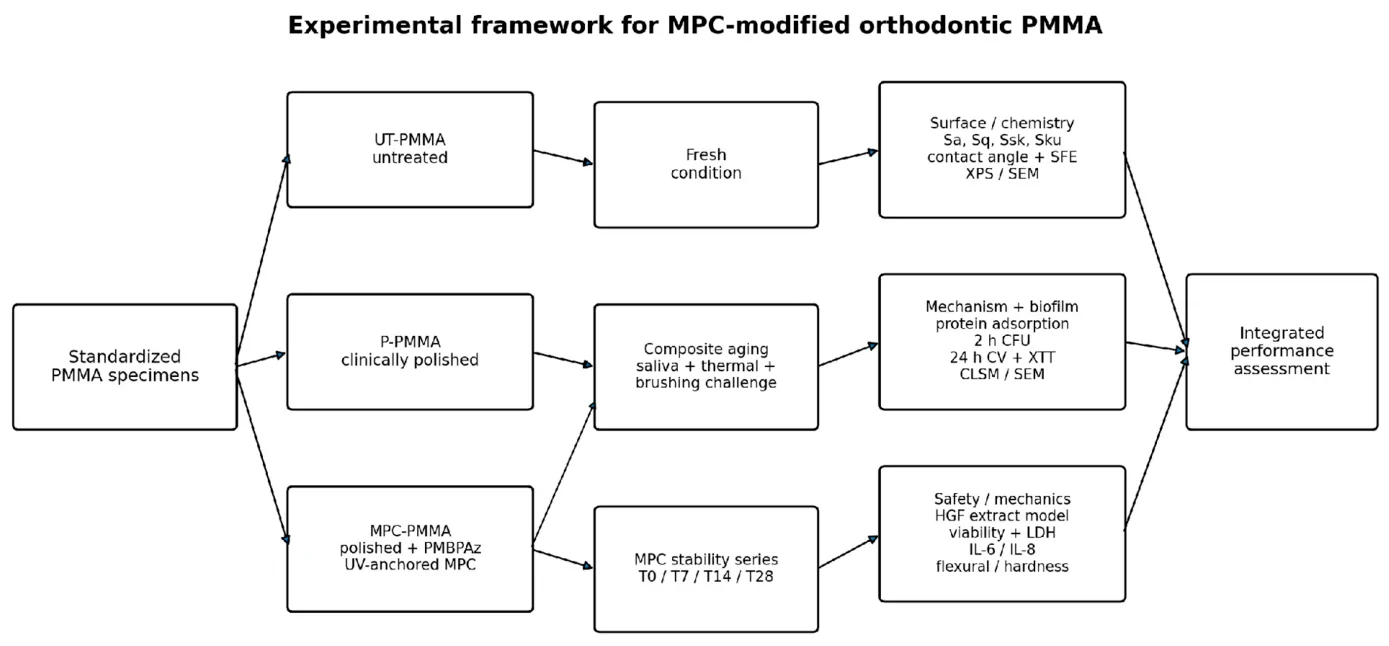
Experimental framework for evaluation of a zwitterionic MPC-modified orthodontic PMMA. UT-PMMA, untreated PMMA; P-PMMA, clinically polished PMMA; MPC-PMMA, polished PMMA coated with photoreactive poly(MPC-co-BMA-co-MPAz).

Figure 1 summarizes the experimental workflow, including the six-condition factorial design, the separate MPC coating-stability series, and the integrated physicochemical, microbiological, mechanical, and cellular endpoints.

### 2.2. Specimen Fabrication, Polishing, and MPC Surface Modification

Heat-polymerizing acrylic PMMA resin (GC Corporation, Tokyo, Japan) was fabricated according to the manufacturer’s processing instructions in Type IV gypsum flasks and finished as 10.00 ± 0.05 mm diameter × 2.00 ± 0.05 mm thick disks (Mitutoyo Absolute Digimatic digital caliper, 0.01 mm resolution). Both faces and the rim were treated; the calculated exposed area was 2.20 cm^2^. After carbide-bur flash removal at 15,000 rpm, all disks were planarized with wet SiC P400 for 30 s per face at 300 rpm and 10 N on a Struers LaboPol-30 (Ballerup, Denmark). UT-PMMA received no further polishing. For P-PMMA and MPC-PMMA, the same operator applied wet SiC P800 and P1200 (each 300 rpm, 10 N, 30 s per face), fine pumice slurry (1:1 with water; felt wheel, 1500 rpm, 60 s per face), and Universal Polishing Paste (Ivoclar Vivadent, Schaan, Liechtenstein; cotton wheel, 1500 rpm, 60 s per face); the rim received the latter two steps for 20 s. Finishing media were renewed every 10 specimens, and polished lots were screened for Sa < 0.2 µm. Different specimens were reserved for destructive tests; technical repeated readings were summarized within each specimen before analysis.

After polishing, specimens were sonicated in deionized water for 10 min, exposed to 70% ethanol for 10 min, air-dried under laminar flow, and sterilized at 254 nm for 30 min per face (UVP CL-1000, Analytik Jena, Upland, CA, USA). The photoreactive PMBPAz copolymer, poly(2-methacryloyloxyethyl phosphorylcholine-co-n-butyl methacrylate-co-2-methacryloyloxyethyl-4-azidobenzoate) [poly(MPC-co-BMA-co-MPAz); MPC/BMA/MPAz molar composition 60:30:10], was synthesized and supplied by the research group of K. Ishihara in Japan. A 0.5 wt% solution of PMBPAz in analytical-grade absolute ethanol was passed through a 0.22 µm PTFE filter. Using a sterile disposable brush, 20 µL was distributed on the specimen face and 10 µL around the rim. The first coat was left for 10 min at room temperature in a closed ethanol vapor-saturated container, after which a second coat was applied using the same procedure. Each face was irradiated at 254 nm for 2 min (UVP CL-1000; session-specific irradiance and UV dose recorded with a UVP UVX/UVX-25 radiometer); the two-coat/UV sequence was carried out on the opposite face in turn. The specimen was then rinsed in 2 mL of sterile deionized water for 5 min and twice more for 2 min. Ethanol was not used after irradiation. Before bacterial experiments, specimens were immersed for 1 h at 37 °C in 1.0 mL of artificial saliva containing 2.5 g/L porcine gastric mucin type II and rinsed once with PBS (pH 7.4).

### 2.3. Simulated Intraoral Aging and Surface-Stability Time Course

Two distinct aging assessments were applied. In the principal 3 × 2 factorial comparison, specimens underwent 28 days in 2 mL of Fusayama–Meyer artificial saliva at 37 °C (NaCl 0.4, KCl 0.4, CaCl_2_·2H_2_O 0.795, NaH_2_PO_4_·H_2_O 0.69, Na_2_S·9H_2_O 0.005, and urea 1.0 g/L; pH 6.8; renewed twice weekly), followed by 5000 thermocycles between 5 ± 1 °C and 55 ± 1 °C (30 s per bath and 10 s transfer; THE-1100, SD Mechatronik) and simulated toothbrushing (ZM-3.8; Oral-B Indicator 35 soft brushes; 2 N, 1.5 Hz, 10 mm stroke, 10,000 bidirectional cycles per face, using a 1:2 w/w Colgate Total–water slurry). Specimens were subsequently sonicated in water for 5 min. All experimental group codes ending in −A designate the final composite-aged condition (saliva immersion, thermocycling, and toothbrushing), not saliva immersion alone. A separate hydrolytic coating-stability series comprised experimentally measured observations at T0, T7, T14, and T28 during artificial saliva immersion only; no intermediate values were interpolated. T0 represents the same fresh, unaged MPC-PMMA experimental condition as MPC-F, explaining their identical baseline summaries, whereas T28 (saliva only) and MPC-A (complete composite aging) are distinct endpoints. The same six individually identified MPC-PMMA disks were followed repeatedly for contact angle, surface free energy, and areal topography, relocating fields using an edge mark. XPS was performed on three independent MPC-PMMA disks at each time point, with two analysis spots per disk (six technical readouts per time point); the disk, not the spot, was the independent experimental unit; coating thickness was evaluated on separately coated silicon reference wafers. Before biological testing, all groups received the same final 254 nm UV exposure (30 min per face) Table 2.

### 2.4. Three-Dimensional Surface Topography

Areal texture was assessed using a Sensofar S neox optical confocal profilometer (Terrassa, Spain; 20× EPI objective, NA 0.45) at five predefined 1 × 1 mm positions per disk (one central and four peripheral fields at 2.5 mm from the center). SensoMAP Premium processing comprised least-squares planar form removal, Gaussian S-filter λs = 2.5 µm, and L-filter 0.25 mm; missing values were interpolated only when <5% of the acquisition was affected. Sa, Sq, Ssk, and Sku were calculated according to ISO 25178-2 [14]. A 5 × 0.5 mm central strip permitted extraction of five 4 mm evaluation profiles for Ra (λc = 0.8 mm; ISO 21920-2 [15]). For atomic force microscopy, a Bruker Dimension Icon in tapping mode (RTESPA-300 silicon cantilever) acquired 20 × 20 µm and 5 × 5 µm images (512 × 512 pixels, 0.8 Hz); first-order flattening and texture assessment were performed in NanoScope Analysis 3.0. According to the confirmed longitudinal protocol, areal-texture readings at T0, T7, T14, and T28 were performed on the same six marked MPC-PMMA disks in predefined relocated fields; multiple fields within a disk were averaged and not considered independent specimens.

### 2.5. Wettability and Surface Free Energy

Static contact angles were measured using a Krüss DSA100 goniometer with ADVANCE software (Hamburg, Germany) at 22 ± 1 °C and 45 ± 10% relative humidity. Five 3 µL sessile droplets of ultrapure water (18.2 MΩ·cm) and five of diiodomethane (≥99%, Sigma-Aldrich) were applied to separate, previously unwetted areas per disk; images were obtained 5 s after deposition, and the contact angles were determined by elliptical fitting. Total, polar, and dispersive surface free-energy components were calculated for each disk from the two-liquid Owens–Wendt–Rabel–Kaelble equation, using water dispersive/polar values of 21.8/51.0 and diiodomethane values of 50.8/0 mN/m. For the hydrolytic stability series, six identifiable MPC-PMMA disks were observed repeatedly at T0, T7, T14, and T28; each disk contributed one mean of its technical droplet measurements at each time point. Hydrated-surface captive-bubble measurements on P-PMMA and MPC-PMMA (3 µL air bubble; 30 s equilibration) were treated separately from sessile-drop measurements.

### 2.6. Surface Chemistry, Morphology, and Coating Integrity

X-ray photoelectron spectroscopy (XPS) was performed using a Thermo Scientific K-Alpha instrument (East Grinstead, UK; monochromatic Al Kα radiation, 1486.6 eV; 400 µm analysis spot; charge neutralization). Survey spectra were recorded over 0–1100 eV (pass energy 200 eV, step 1 eV), with high-resolution C 1s, O 1s, N 1s, and P 2p scans at 20 eV pass energy and 0.1 eV steps; binding energies were calibrated to C 1s at 284.8 eV, and spectra were processed using a Shirley background and Avantage sensitivity factors. For the T0/T7/T14/T28 hydrolytic assessment, XPS used three independent MPC-PMMA disks per time point, with two analysis spots per disk, yielding six technical analytical readouts per time point. The two spot measurements on each disk were technical replicates; the six reported XPS readouts at each time point therefore came from three independent specimens, not six. Descriptive summaries were calculated without treating analytical spots as independent experimental units. XPS samples were distinct from the six disks examined longitudinally for wettability and topography. ATR-FTIR was performed on a Thermo Nicolet iS50 with a Ge ATR crystal (4000–650 cm^−1^, 4 cm^−1^ resolution, and 64 scans), comparing polished PMMA, fresh and aged coated PMMA, and UV-treated and untreated PMBPAz reference films. Bands potentially masked by bulk PMMA were interpreted cautiously. Surface morphology was evaluated using a Hitachi SU5000 FE-SEM (5 kV, 500×/2000×/10,000×), with approximately 5 nm Au/Pd coating and five predefined imaging fields per specimen.

Film thickness was evaluated by spectroscopic ellipsometry on parallel-coated silicon reference wafers (J.A. Woollam M-2000; Cauchy model; three measurement positions per wafer) using two independent wafers at each hydrolytic time point. These wafer measurements represent coating-reference thickness and cannot be interpreted as a direct cross-sectional PMMA-disk film thickness. The wafer was the independent measurement unit, not the individual ellipsometry point. Coating persistence was assessed from complementary chemical, wetting, and microbiological endpoints; XPS elemental signatures alone do not establish microscopic film uniformity or continuity.

### 2.7. Protein Adsorption as a Mechanistic Anti-Fouling Endpoint

A separate set of matched-by-batch twin disks was allocated to adsorption and bacterial-adhesion assays because protein extraction and bacterial detachment are destructive. For the protein assay, disks were incubated for 1 h at 37 °C with 1.0 mL of 1 mg/mL bovine serum albumin (fraction V, Sigma-Aldrich A7030) in PBS (pH 7.4), washed three times with PBS, and transferred to clean wells. Adsorbed material was recovered with 1.0 mL of 1% SDS in PBS (30 min at 37 °C, 100 rpm, and then a 10 min bath sonication) and quantified using the Micro BCA Protein Assay Kit (Thermo Fisher 23235; BSA standards 0–40 µg/mL in the SDS matrix, 150 µL of sample plus 150 µL of reagent, 2 h at 37 °C; OD562). Material blanks were subtracted, and the mass was divided by the 2.20 cm^2^ exposed area. Twin disks shared a batch/pair identifier but were not the same physical specimen; pairing does not establish within-disk mechanistic causality.

### 2.8. Mechanical Properties

Flexural testing was performed on independent 64.0 ± 0.2 × 10.0 ± 0.2 × 3.3 ± 0.2 mm PMMA bars (10 per experimental condition) after 50 ± 2 h conditioning in 37 ± 1 °C water, using an Instron 5967 machine (1 kN cell; Bluehill Universal) in three-point bending with a 50.0 ± 0.1 mm support span, 3.2 mm support/punch radii, and 5 ± 1 mm/min crosshead speed until failure. Flexural strength (MPa) was calculated as σ = 3Fl/(2bh^2^), with F being the maximum load, l being the span, b being the width, and h being the thickness. The specified specimen geometry, conditioning, loading configuration, and equation were taken from the relevant provisions of ISO 20795-1:2013 [16]; the additional artificial-aging sequence and applied surface finish/coating were study-specific modifications, not claims of full ISO conformity. Vickers microhardness (Shimadzu HMV-G, 0.49 N, 15 s) was measured by five separate impressions per disk (six disks per condition), using ISO 6507-1:2023 [17] only for general indentation geometry and calculations. Because the indentation penetrates beyond the thin surface coating, the Vickers results primarily reflect the substrate.

### 2.9. Streptococcus mutans Strain and Culture Conditions

*Streptococcus mutans* UA159 (ATCC 700610; Manassas, VA, USA) was revived from −80 °C BHI/glycerol stock and cultured in Bacto Brain Heart Infusion broth and BHI agar (BD, Franklin Lakes, NJ, USA) at 37 °C under 5% CO_2_. Overnight cultures (16–18 h) were diluted 1:20 into fresh BHI and grown to OD600 ≈ 0.50, with session-specific viable counts verified by plating. Early adhesion used 1 × 10^6^ CFU/mL in sucrose-free BHI; the 24 h biofilm assays used 1 × 10^7^ CFU/mL in BHI containing 1% *w*/*v* sucrose. Specimens were preconditioned for 1 h at 37 °C in artificial saliva with mucin, as described in Section 2.2.

### 2.10. Early Bacterial Adhesion

Conditioned disks in individual 24-well positions were incubated with 1.0 mL of bacterial suspension for 2 h at 37 °C and 5% CO_2_. Non-adherent organisms were removed by three 1.0 mL PBS washes without direct fluid impingement on the surface. Each disk was transferred to 1.0 mL of PBS; adherent bacteria were released by vortexing for 60 s (Vortex-Genie 2), bath sonication at ~40 kHz for 5 min (Bransonic M1800), and another 30 s vortex. Serial 1:10 dilutions were plated in duplicate (100 µL) on BHI agar and incubated for 48 h. Plates containing 30–300 colonies were used to calculate CFU per 2.20 cm^2^ exposed area and log10-transformed for analysis. Independent disks, not duplicate plates, defined biological n; sterile specimen and medium-only negative controls accompanied each run.

### 2.11. Biofilm Biomass, Metabolic Activity, and Microscopy

Biofilms were generated on separate disks for 24 h in 1.0 mL of BHI plus 1% sucrose. Crystal violet biomass was determined after three PBS washes and transferred to clean wells; 15 min fixation with absolute methanol, 15 min air drying, staining with 1.0 mL of 0.1% crystal violet (Sigma-Aldrich C0775) for 15 min, three water washes, and extraction with 1.0 mL of ethanol/isopropanol (1:1) for 15 min at 100 rpm. An aliquot of 200 µL was read at OD590 after subtraction of a material-specific sterile-disk blank. On different disks, XTT (Sigma-Aldrich X4626, 1 mg/mL) plus menadione (Sigma-Aldrich M5625, 1 µM) was added in 500 µL of PBS for 2 h at 37 °C in darkness; 200 µL of supernatant was read at 450 nm (630 nm reference). Optical readings were obtained using a BioTek Synergy H1 (Agilent Technologies, Santa Clara, CA, USA). All endpoint-specific technical readings were aggregated per independent disk.

Confocal biofilm imaging used the LIVE/DEAD BacLight viability kit (Thermo Fisher L7012; SYTO 9 plus propidium iodide; 15 min dark incubation) and a Leica Stellaris CLSM (488/561 nm excitation with separated emission channels). Z-stacks were acquired with a 40× water-immersion objective, 1024 × 1024 pixels, and 1 µm Z step from five fields on each of the three disks per condition; biofilm thickness and biovolume were calculated by COMSTAT2 in Fiji using a common Otsu threshold and disk-level aggregation. Complementary SEM biofilms were fixed with 2.5% glutaraldehyde in 0.1 M sodium cacodylate (pH 7.4), dehydrated in ascending ethanol, dried with hexamethyldisilazane, sputter-coated with ~5 nm Au/Pd, and examined on the Hitachi SU5000 at 5 kV (1000×–10,000×). SEM was a qualitative structural endpoint.

### 2.12. Gingival Fibroblast Model, Cytocompatibility, and Inflammatory Response

Human gingival fibroblasts (HGF-1, ATCC CRL-2014; Manassas, VA, USA) were cultured in high-glucose DMEM containing GlutaMAX and pyruvate (Gibco 31966-021), 10% fetal bovine serum (Gibco 10270-106), and 100 U/mL penicillin/100 µg/mL streptomycin (Gibco 15140-122) at 37 °C and 5% CO_2_. Cells between passages 6–12, after receipt, were used, with a seeding density of 2.5 × 10^4^ cells/well in 48-well plates and 24 h attachment before treatment. An extract-based approach was used to assess the soluble effects rather than direct attachment to an anti-fouling surface. The extraction ratio, exposure, and cytotoxicity threshold followed specified elements of ISO 10993-5:2009 [18] (cytotoxicity threshold) and ISO 10993-12:2021 [19] and its 2025 amendment [20] (extract preparation); use of HGF-1 and WST-8 was a dental-context adaptation, not a claim to have reproduced every ISO-prescribed reference-cell procedure.

Fresh and composite-aged materials were extracted as two disks (4.40 cm^2^ total exposed surface) per 1.47 mL of complete medium (3 cm^2^/mL) at 37 ± 1 °C for 24 ± 2 h in closed sterile polypropylene tubes. Six independently prepared extracts per condition (in three experimental sessions) were tested at 100% and 50%; each well received 250 µL of extract for 24 h. The following controls were included: untreated cells in fresh complete medium; extraction medium incubated without material; extracted HDPE reference material as the negative material control; 0.1% ZDEC polyurethane extract as the positive cytotoxic material control; maximum-lysis buffer for LDH; and 1 ng/mL recombinant human IL-1β for the cytokine-stimulation control. All were processed under the relevant assay conditions. For viability, supernatant was removed, and 10% *v*/*v* CCK-8/WST-8 reagent (Dojindo CK04) was added for 2 h; OD450 (650 nm reference) was blank-adjusted and expressed relative to the untreated cell reference. The ISO 10993-5 quantitative 70% viability threshold was used as a cytotoxicity screening criterion.

From the corresponding 24 h cell-exposure wells, 50 µL of supernatant was subjected to the CyQUANT LDH Cytotoxicity Assay (Thermo Fisher C20300; 50 µL of the reaction mixture for 30 min at room temperature; 490 nm, 680 nm reference). LDH release was expressed relative to spontaneous release and complete lysis controls. Remaining supernatants were centrifuged (300 *g*, 5 min), frozen at −80 °C, and later used for human IL-6 and IL-8 sandwich ELISAs (R&D Systems DuoSet DY206 and DY208, with DY008 reagents; duplicate measures and seven-point four-parameter calibration; 450 nm and 570 nm correction; IL-8 diluted 1:5). The IL-6 and IL-8 concentrations are presented in pg/mL without normalization to cell viability. The endotoxin-screening protocol used the Pierce Chromogenic Endotoxin Quant Kit (Thermo Fisher A39552). These cytokine readouts characterize cell response to material extracts under laboratory conditions, not clinical mucosal inflammation.

### 2.13. Experimental Controls, Randomization, and Blinding

Negative microbiological controls comprised sterile specimens undergoing the full processing sequence without *S. mutans*, alongside medium-only wells and plated inoculum checks. For cell experiments, untreated cell, extraction medium, HDPE-negative, ZDEC-positive, maximum lysis, and IL-1β-positive reference conditions were used as specified in Section 2.12. Sample allocation and plate positions were randomized by session, and image/assay analyses used coded specimen identifiers when feasible. A matched sham-treated polished disk exposed to coating solvent, UV, and rinsing (but no polymer) was not included; this is acknowledged as a limitation.

### 2.14. Sample Size and Replication

The primary bacterial adhesion endpoint comprised 12 independent disks per surface × aging condition (total n = 72); technical readings were averaged within each specimen. An SD of approximately 0.32 log10 CFU/cm^2^ in prior work informed a target difference of 0.50 log10 CFU/cm^2^ at 90% power. In the separate saliva-only hydrolytic stability series, the same six MPC-PMMA disks were repeatedly measured at T0, T7, T14, and T28 for the water contact angle, surface free energy, and topography. XPS involved three independent disks per time point, with two analysis spots per disk, giving six technical readouts per time point. The experimental unit is the disk, not the measurement spot. Thickness was evaluated on separately coated silicon reference wafers, which are distinct from the PMMA disks. T0 was equivalent to the fresh MPC-F condition; groups ending in −A received all three aging challenges.

### 2.15. Statistical Analysis

The primary 2 h adhesion endpoint (log10 CFU/cm^2^) was evaluated in a two-factor surface condition (UT, P, MPC) × aging (fresh, composite-aged) linear model, with an experimental session included as a blocking factor. The prespecified MPC-versus-P contrasts were evaluated separately within fresh and fully composite-aged states, with Holm adjustment for the two planned comparisons; secondary pairwise comparisons were adjusted within endpoint families. Percentage reductions were calculated as 100 × [1 − 10^(mean log10 MPC − mean log10 P)]. Distributional and variance assumptions were examined using residual plots, as well as Shapiro–Wilk and Levene assessments. Other independent-disk endpoints were analyzed within the factorial framework. In the separate T0/T7/T14/T28 saliva-only stability series, wettability, surface free energy, and topography were measured repeatedly on six disks; time-dependent findings are presented descriptively; formal inference on these repeated measures would require a mixed-effects model incorporating disk identity, and no unverified longitudinal *p*-values are reported. XPS measurements were obtained from three independent disks per time point, with two analysis spots per disk (with six technical analytical readouts per time point). The laboratory-reported mean ± SD XPS summaries describe the available analytical readings and must not be interpreted as n = 6 independent specimens or as confirmed between-disk variability; no formal XPS time-course significance tests were derived. Measurements on silicon reference wafers were evaluated separately. The saliva-only T28 measurements were not pooled with or substituted for MPC-A observations after composite aging.

For gingival-cell outcomes, each independently prepared extract, rather than an assay well or optical readout, was the statistical unit (six extracts per condition). Dunnett-adjusted contrasts compared each material condition with untreated-cell controls, while factorial and planned contrasts assessed the material surface condition, aging, and MPC-versus-P differences. Nonsignificant MPC-versus-P findings were not interpreted as proof of equivalence; effect estimates, variability, and control-cell comparisons informed the interpretation of viability, LDH, and inflammatory mediators.

Exploratory protein–adhesion analyses used the paired identifiers linking separate batch-matched twin disks after averaging technical measurements within each disk. The pooled Pearson correlation was evaluated alongside surface group-adjusted and batch-adjusted partial associations and mixed-effects regression, including preparation batch. A cluster-robust sensitivity analysis assessed the stability of the protein coefficient across the six batches. Given the small number of batches and collinearity between adsorption and surface treatment, these associations were regarded as exploratory and not evidence of causal mediation.

### 2.16. Primary and Secondary Outcomes

The prespecified primary outcome was an *S. mutans* adherent load after 2 h, expressed as log10 CFU/cm^2^. Key secondary outcomes were 24 h biofilm biomass (CV OD590), 24 h metabolic activity (XTT OD450), and retention of the MPC-associated anti-biofilm effect after aging. Mechanistic secondary outcomes were protein adsorption, water contact angle, surface free energy, and persistence of the PMBPAz chemical signature by XPS. Additional outcomes included 3D topography, biofilm thickness/biovolume, microhardness/flexural strength, gingival fibroblast viability, LDH release, and IL-6/IL-8 production.

### 2.17. Ethical Considerations

This in vitro study used an established commercially available human gingival fibroblast cell line (HGF-1, ATCC CRL-2014). No participants were recruited, no new human tissue was collected, and no animal experiments were performed. Consequently, study-specific ethics committee approval was not required for the experiments described.

## 3. Results

### 3.1. Experimental Overview and Baseline Surface Characteristics

For the primary microbiological endpoint, 72 independent specimens were included in the final analysis (n = 12 per surface × aging condition). Two additional specimens were excluded because of visible surface defects that were identified at baseline inspection according to prespecified criteria. Baseline characterization confirmed the intended distinction between UT-PMMA and P-PMMA and verified successful formation of the PMBPAz-MPC surface on MPC-PMMA through characteristic XPS phosphorus/nitrogen signals together with a marked reduction in the water contact angle.

The six-group surface summaries in Table 3 represent fresh (−F) and fully composite-aged (−A) conditions; thus MPC-A must not be equated with the saliva-only T28 endpoint reported separately in Table 4. Where baseline and aged values derive from repeated assessment of the same specimen, within-disk correlation must be accounted for in inferential analyses; the independent-disk microbiological model cannot be applied unchanged to these observations.

**Table 3:** Surface properties of PMMA groups before and after simulated intraoral aging.

Group	Sa (µm)	Sq (µm)	Ssk	Sku	Water Contact Angle (°)	Surface Free Energy (mN/m)
UT-F	0.42 ± 0.08	0.55 ± 0.10	0.31 ± 0.15	3.8 ± 0.6	78.5 ± 4.2	38.2 ± 3.1
UT-A	0.48 ± 0.09	0.62 ± 0.11	0.35 ± 0.16	4.0 ± 0.7	81.3 ± 4.5	36.0 ± 3.4
P-F	0.11 ± 0.03	0.15 ± 0.04	0.08 ± 0.10	3.1 ± 0.4	71.4 ± 3.6	41.5 ± 2.8
P-A	0.14 ± 0.03	0.18 ± 0.04	0.10 ± 0.11	3.2 ± 0.4	74.8 ± 3.9	39.7 ± 3.0
MPC-F	0.12 ± 0.03	0.16 ± 0.04	0.07 ± 0.09	3.0 ± 0.4	18.6 ± 2.7	61.3 ± 3.5
MPC-A	0.13 ± 0.03	0.17 ± 0.04	0.08 ± 0.10	3.1 ± 0.4	24.3 ± 3.1	57.8 ± 3.7

**Table 4:** Saliva-only hydrolytic stability of MPC-PMMA at experimentally measured T0, T7, T14, and T28. The baseline T0 condition is equivalent to fresh MPC-F; T28 is distinct from the fully composite-aged MPC-A group reported in Table 3 and Table 5.

Time	n (Disks) †	Water Angle (°)	SFE (mN/m)	Sa (µm)	XPS P (at.%)	XPS N (at.%)
T0	6	18.6 ± 2.7	61.3 ± 3.5	0.12 ± 0.03	3.8 ± 0.3	2.1 ± 0.2
T7	6	20.1 ± 2.8	60.4 ± 3.6	0.12 ± 0.03	3.6 ± 0.3	2.0 ± 0.2
T14	6	21.0 ± 2.9	59.8 ± 3.6	0.12 ± 0.03	3.4 ± 0.3	1.9 ± 0.2
T28	6	21.7 ± 3.0	59.2 ± 3.7	0.12 ± 0.03	3.3 ± 0.4	1.8 ± 0.3

**Table 5:** Surface chemical signatures, coating thickness on reference silicon wafers, protein adsorption, and mechanical outcomes in fresh (−F) and fully composite-aged (−A) groups. The MPC-A values do not represent saliva-only T28.

Outcome	UT-F	UT-A	P-F/P-A	MPC-F	MPC-A
XPS P/N surface signal (at.%)	not detected	not detected	not detected/not detected	3.8% P/2.1% N	2.9% P/1.6% N
Wafer coating thickness (µm)	N/A	N/A	N/A	0.18 ± 0.04	0.15 ± 0.05
Vickers microhardness (HV)	18.9 ± 1.4	18.2 ± 1.6	19.1 ± 1.3/18.6 ± 1.5	18.8 ± 1.4	18.3 ± 1.5
Flexural strength (MPa)	84.6 ± 5.7	80.1 ± 6.2	85.3 ± 5.4/81.7 ± 5.9	84.9 ± 5.6	81.2 ± 6.0
Protein adsorption (µg/cm^2^)	4.85 ± 0.62	5.10 ± 0.58	4.20 ± 0.55/4.55 ± 0.60	1.35 ± 0.31	1.72 ± 0.38

### 3.2. Chemical Persistence and Coating Integrity

At baseline, the fresh MPC-PMMA condition (T0, equivalent to MPC-F) showed a water contact angle of 18.6 ± 2.7°, surface free energy of 61.3 ± 3.5 mN/m, and XPS signals of 3.8 ± 0.3 at.% P and 2.1 ± 0.2 at.% N. During the separate saliva-only immersion series, the contact angle increased descriptively to 20.1 ± 2.8° at T7, 21.0 ± 2.9° at T14, and 21.7 ± 3.0° at T28; surface free energy decreased to 59.2 ± 3.7 mN/m at T28, while Sa remained approximately 0.12 ± 0.03 µm throughout. The corresponding XPS signals at T28 were 3.3 ± 0.4 at.% P and 1.8 ± 0.3 at.% N (Table 4). By contrast, the distinct MPC-A condition underwent additional thermocycling and simulated brushing after the 28-day saliva immersion and showed a larger descriptive wettability/chemical change: a contact angle of 24.3 ± 3.1°, surface free energy of 57.8 ± 3.7 mN/m, Sa of 0.13 ± 0.03 µm, and P/N signals of 2.9/1.6 at.% (Table 3 and Table 5). The changes were thus directionally greater after composite aging than after saliva immersion alone; however, no direct between-protocol statistical test is claimed. XPS confirms persistence of coating-associated elemental surface signals but does not demonstrate uninterrupted film continuity or the absence of microscopic defects. For the time course, the XPS spread is descriptive from technical readouts and does not represent six independent disk-level replicates.

Note: Water contact angle, surface free energy, and Sa are means ± SDs from six individual MPC-PMMA disks measured longitudinally at each time point (n = 6 disks). XPS was performed on three independent disks per time point, with two analysis spots per disk (six technical analytical readouts per time point). XPS mean ± SD values describe the available analytical readouts; the corresponding SDs must not be interpreted as between-disk SDs, and no inferential test used the six technical readouts as independent observations. All four time points were measured experimentally, without interpolation. No wafer-thickness time-course data are tabulated here. T0 and MPC-F denote the same unaged condition; T28 denotes saliva-only immersion, whereas MPC-A reflects saliva followed by thermocycling and brushing.

### 3.3. Mechanical Properties

Microhardness and flexural strength remained closely comparable between P-PMMA and MPC-PMMA. In the fresh state, the Vickers microhardness was 19.1 ± 1.3 for P-PMMA and 18.8 ± 1.4 for MPC-PMMA, while flexural strength was 85.3 ± 5.4 MPa and 84.9 ± 5.6 MPa, respectively. After aging, the corresponding values were 18.6 ± 1.5 versus 18.3 ± 1.5 for microhardness and 81.7 ± 5.9 MPa versus 81.2 ± 6.0 MPa for flexural strength, indicating no meaningful deterioration associated with the surface coating.

### 3.4. Protein Adsorption

Protein adsorption was substantially lower on MPC-PMMA than on P-PMMA in the fresh condition (1.35 ± 0.31 vs. 4.20 ± 0.55 µg/cm^2^; adjusted *p* < 0.0001), corresponding to a 67.9% reduction. After aging, the corresponding values were 1.72 ± 0.38 and 4.55 ± 0.60 µg/cm^2^ (adjusted *p* < 0.0001), corresponding to a 62.2% reduction. The surface × aging interaction was significant (*p* = 0.041), indicating modest attenuation but persistence of the anti-fouling effect.

### 3.5. Early S. mutans Adhesion

Early bacterial adhesion differed significantly among surface conditions (surface main effect, *p* < 0.0001). Aging produced a significant main effect (*p* = 0.003), and the surface × aging interaction was significant (*p* = 0.017). In the fresh condition, MPC-PMMA showed a 0.73 log10 CFU/cm^2^ lower adhesion than P-PMMA, corresponding to an 81% reduction (adjusted *p* < 0.0001). After aging, the difference remained 0.62 log10 CFU/cm^2^, corresponding to a 76% reduction (adjusted *p* < 0.0001). Thus, the anti-adhesive effect was attenuated but remained substantial after aging. Polishing alone also reduced early adhesion: P-PMMA was 0.43 log10 CFU/cm^2^ lower than UT-PMMA in the fresh condition (adjusted *p* = 0.002).

### 3.6. Biofilm Biomass, Metabolic Activity, and Microscopy

At 24 h, the biofilm biomass was lower on MPC-PMMA than on P-PMMA in both fresh and aged conditions. Relative to P-PMMA, the reduction was 44.6% in the fresh state (0.51 vs. 0.92 OD590) and 37.9% after aging (0.64 vs. 1.03 OD590). The surface effect was significant (*p* < 0.0001), as were the aging effect (*p* = 0.008) and surface × aging interaction (*p* = 0.029). XTT metabolic activity showed the same pattern, with reductions of 51.3% before aging and 43.2% after aging. CLSM showed markedly thinner biofilms on MPC-PMMA: 16.4 ± 3.7 µm for MPC-F and 20.2 ± 4.1 µm for MPC-A, compared with 33.8 ± 5.2 µm for P-F and 37.1 ± 5.6 µm for P-A. Biovolume followed the same trend (Table 6).

### 3.7. Gingival Fibroblast Cytocompatibility and Inflammatory Response

**Figure 2:**
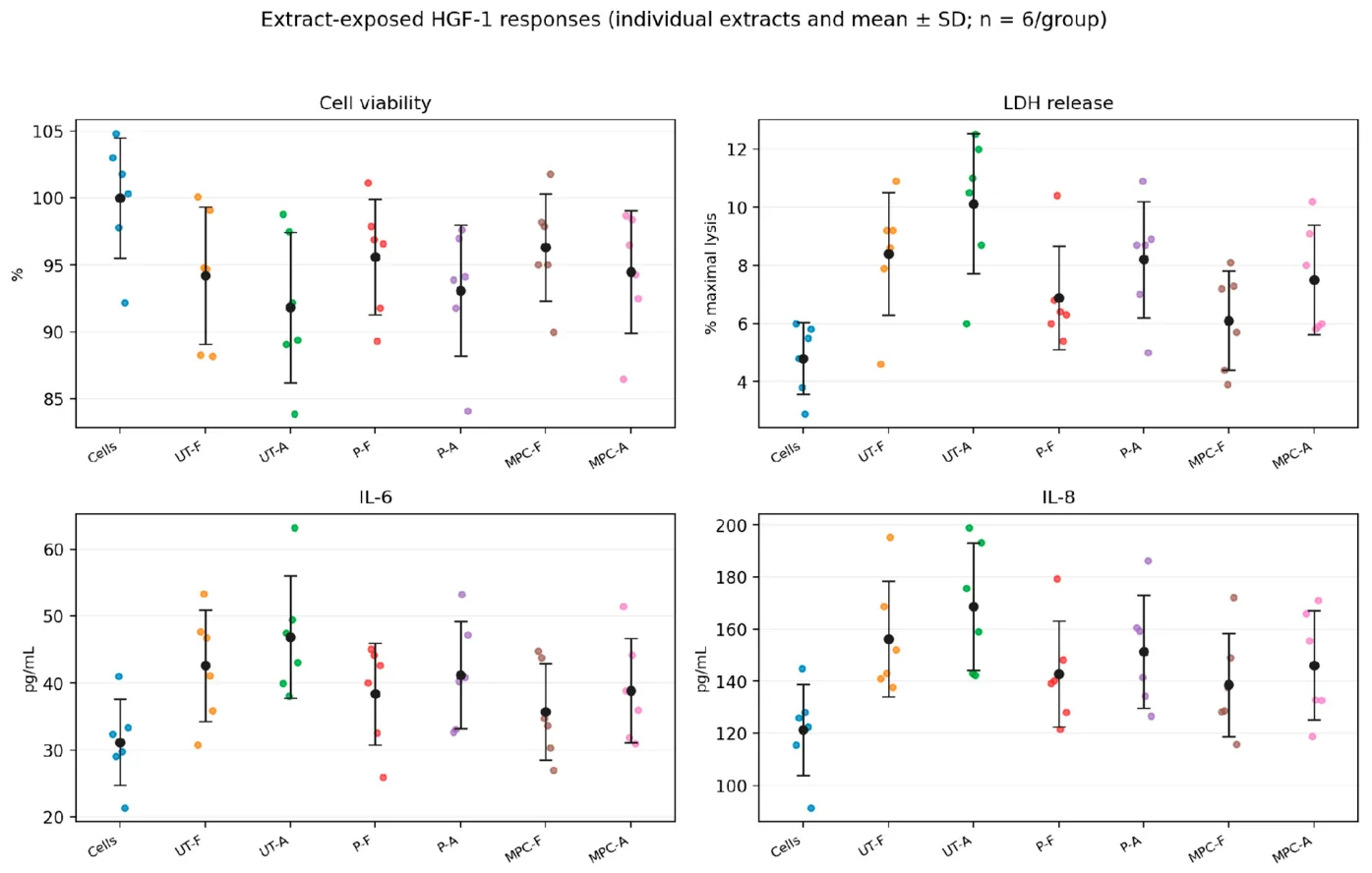
HGF-1 cellular responses to fresh and aged PMMA extracts. Individual values and group means ± SD are shown for six independent extracts per condition; control represents untreated cells. Dunnett-adjusted significance results are reported in Table 7 and Section 3.7.

After 24 h of exposure to the PMMA extracts, the mean gingival fibroblast viability (Figure 2) was 94.2 ± 5.1% for UT-F, 91.8 ± 5.6% for UT-A, 95.6 ± 4.3% for P-F, 93.1 ± 4.9% for P-A, 96.3 ± 4.0% for MPC-F, and 94.5 ± 4.6% for MPC-A (n = 6 independent extracts per group); viability in the untreated-cell control was 100.0 ± 4.5%. Compared with untreated cells, UT-A showed a reduction in viability (Dunnett-adjusted *p* = 0.025). LDH release was higher for UT-F (*p* = 0.012), UT-A (*p* < 0.001), and P-A (*p* = 0.019), whereas the corresponding comparisons for MPC-F (*p* = 0.701) and MPC-A (*p* = 0.086) were not statistically significant. IL-6 was elevated in UT-A (*p* = 0.007), and IL-8 was elevated in UT-F (*p* = 0.033) and UT-A (*p* = 0.002) relative to the untreated cells. The planned comparisons between MPC-PMMA and P-PMMA under the same aging condition did not show statistically significant differences in viability, LDH, IL-6, or IL-8. These findings indicate measurable extract-associated effects in selected groups but do not establish equivalence between coated and polished materials or clinical biocompatibility.

**Table 7:** Human gingival fibroblast responses to fresh and aged PMMA extracts, with comparisons against the untreated-cell control. The final row summarizes assay-specific positive-reference results, not a single common positive-control exposure across all four endpoints.

Group	Cell Viability (%)	LDH Release (% of Maximal Lysis)	IL-6 (pg/mL)	IL-8 (pg/mL)	Interpretation vs. Control
UT-F	94.2 ± 5.1	8.4 ± 2.1	42.6 ± 8.3	156.3 ± 22.1	NS (viability); LDH *p* = 0.012; IL-8 *p* = 0.033
UT-A	91.8 ± 5.6	10.1 ± 2.4	46.9 ± 9.1	168.7 ± 24.5	Viability *p* = 0.025; LDH *p* < 0.001; IL-6 *p* = 0.007; IL-8 *p* = 0.002
P-F	95.6 ± 4.3	6.9 ± 1.8	38.4 ± 7.6	142.8 ± 20.3	No endpoint significant vs. cells in Dunnett-adjusted analyses
P-A	93.1 ± 4.9	8.2 ± 2.0	41.2 ± 8.0	151.4 ± 21.7	LDH *p* = 0.019 vs. cells
MPC-F	96.3 ± 4.0	6.1 ± 1.7	35.7 ± 7.2	138.6 ± 19.8	No endpoint significant vs. cells in Dunnett-adjusted analyses
MPC-A	94.5 ± 4.6	7.5 ± 1.9	38.9 ± 7.8	146.2 ± 20.9	No endpoint significant vs. cells in Dunnett-adjusted analyses
Untreated cell control	100.0 ± 4.5	4.8 ± 1.2	31.2 ± 6.4	121.4 ± 17.6	Reference: measured 100.0 ± 4.5% viability
Assay-specific positive references	22.4 ± 6.3	88.7 ± 6.5	118.5 ± 15.2	412.8 ± 38.6	Different positive controls were used for cytotoxicity and cytokine assays; see Section 2.12.

### 3.8. Relationships Between Surface Properties and Biological Outcomes

Protein adsorption and early bacterial adhesion were evaluated on separate, batch-matched twin disks (36 linked pairs across six manufacturing batches and six surface-by-aging conditions), with technical readings averaged within each assay specimen. The pooled association across pairs was positive (Pearson r = 0.834, *p* < 0.001), although it was influenced by between-group differences and cannot be interpreted as a within-disk mechanism. After adjustment for experimental group and preparation batch, the partial association was smaller (r = 0.428; 95% CI, 0.049–0.699; *p* = 0.029). A mixed-effects model incorporating a batch random intercept yielded an adsorption coefficient of 0.156 log10 CFU/cm^2^ per µg/cm^2^ adsorbed protein (95% CI, 0.012–0.300; *p* = 0.035). Nevertheless, substantial collinearity with the experimental surface condition (with a variance inflation factor of approximately 11.1) and a nonsignificant batch-clustered sensitivity analysis (*p* = 0.620) indicated that the adjusted association was not robust. These exploratory analyses support co-variations in anti-fouling and anti-adhesive responses across groups but do not establish protein adsorption as an independent causal mediator.

## 4. Discussion

The present study extends our previous comparative investigation by moving from identification of a high-colonization orthodontic material to targeted engineering of that same material. The principal finding was that photoreactive PMBPAz-MPC modification produced a marked reduction in early *S. mutans* adhesion and 24 h biofilm formation compared with clinically polished PMMA, and a substantial proportion of this effect persisted after composite simulated intraoral aging. In the fresh state, MPC-PMMA reduced early adhesion by 0.73 log10 CFU/cm^2^ (81%) and biofilm biomass by 44.6% relative to polished PMMA. After aging, the corresponding reductions remained 0.62 log10 CFU/cm^2^ (76%) and 37.9%. These microbiological effects were accompanied by reduced protein adsorption, persistence of MPC-associated XPS signals, and pronounced hydrophilization, with broadly preserved mechanical outcomes and a more nuanced extract-based cellular response requiring comparisons with untreated cells. These findings may be relevant to periodontal considerations in orthodontics, but clinical periodontal benefits were not evaluated in this in vitro study [21].

The experimental groups shared the same heat-cured PMMA substrate, enabling a clinically relevant comparison between polishing alone and polishing followed by PMBPAz-MPC coating. However, P-PMMA was not a matched sham-treated control: without a solvent/UV/rinse-only arm, not all aspects of the coating application process can be distinguished from polymer-specific effects. Thus, the coating-associated differences are meaningful within this design but cannot be assigned exclusively to the zwitterionic chemical groups.

The microbiological findings directly address the hypothesis generated by our previous work, in which PMMA showed the greatest early *S. mutans* adhesion and biofilm biomass among the orthodontic materials tested [1]. In the present study, targeted modification of that PMMA surface reduced its colonization potential well beyond the effect achieved by polishing alone. The concordant reductions in CFU, crystal violet biomass, XTT metabolic activity, CLSM biofilm thickness, and biovolume indicate that the intervention affected both initial attachment and subsequent early biofilm development rather than a single assay-specific endpoint.

PMBPAz-mediated surface modification of dental PMMA and reduction in plaque deposition have been demonstrated previously [2,3,4,5]. The present contribution lies in applying an existing material strategy to an orthodontic PMMA substrate under standardized polishing and a multi-endpoint evaluation, including composite aging, surface chemistry, protein adsorption, bacterial outcomes, flexure/hardness, and gingival-fibroblast extract responses. The data should therefore be framed as an integrated orthodontic-specific confirmation and extension, not the first report of PMBPAz coating or its anti-fouling activity.

Fresh polished and coated specimens had closely comparable Sa measurements (0.11 ± 0.03 and 0.12 ± 0.03 µm) despite marked differences in the water contact angle (71.4 ± 3.6° and 18.6 ± 2.7°) and bacterial adhesion. This pattern is compatible with an interfacial-chemistry contribution that is not reducible to areal roughness alone. However, protein and bacterial endpoints used batch-matched twin disks rather than the same disk; pooled correlation across experimental groups is confounded by the intervention itself. After group and batch adjustment, the association was less pronounced, and sensitivity to model assumptions prevents any strong mediation claim. Comparable associations between surface characteristics, early bacterial adhesion, and biofilm formation have been reported for dental resin-based materials, aligner polymers, and orthodontic substrates [22,23,24,25].

Wettability, surface free energy, and protein adsorption provided a coherent mechanistic pattern. The MPC-rich surface reduced the fresh-state contact angle from 71.4° on polished PMMA to 18.6° and increased surface free energy from 41.5 to 61.3 mN/m. At the same time, adsorbed protein decreased from 4.20 to 1.35 µg/cm^2^, a 67.9% reduction. During immersion in artificial saliva alone, the coated-surface contact angle increased only to 21.7° after 28 days, with phosphorus decreasing from 3.8 to 3.3 at.% and nitrogen from 2.1 to 1.8 at.%; Sa remained at approximately 0.12 µm. The separate composite-aged endpoint, which added thermocycling and brushing, showed a larger descriptive shift in contact angle (24.3°), phosphorus (2.9 at.%), and nitrogen (1.6 at.%), and a small increase in Sa (0.13 µm). After composite aging, protein adsorption on MPC-PMMA increased to 1.72 µg/cm^2^ yet remained lower than on the corresponding polished control. These distinct exposure conditions support a graded descriptive pattern of coating-associated surface changes, without establishing which added module caused the difference or implying a statistically tested direct comparison of the two aging protocols [26,27].

The two aging assessments address different questions. The longitudinal, saliva-only T0–T28 series quantified hydrolytic changes using repeated measurements on six MPC-PMMA disks for wettability and topography, and separate XPS disks at each time point. T0 coincides with the MPC-F fresh condition; T28 is not synonymous with MPC-A. The composite-aged factorial endpoint added thermocycling and simulated toothbrushing and produced a larger descriptive decline in coating-associated XPS elemental signals than saliva immersion alone. This observation is compatible with an additional surface challenge but cannot isolate the contribution of thermocycling from brushing, and a direct inferential comparison of the two exposure protocols was not performed. Persistence of anti-adhesive effects after the complete challenge supports in vitro functional durability, not an estimate of clinical service life; elemental signals do not prove microscopic film continuity or detailed biofilm architecture.

A clinical functional evaluation of interceptive orthodontic appliances provides context for potential acrylic-appliance applications, without implying that the present in vitro outcomes predict clinical performance [28]. A broader dental materials literature provides additional context for these findings. Studies on orthodontic wires have examined how fluoride exposure, corrosion, water immersion, and esthetic coatings can affect cytocompatibility, surface behavior, and mechanical properties [29,30,31,32,33]. Other investigations have evaluated biofilm modulation and microbial adhesion in relation to antimicrobial agents, orthodontic material surface characteristics, resin composition, surface roughness, and denture-lining materials [34,35,36,37,38,39]. Contact angle, wettability, and surface energy studies further support the relevance of interfacial properties when interpreting biological and adhesive interactions with dental materials [40,41,42]. In orthodontic bonding models, both bonding procedures and composite selection have also been investigated in relation to initial mutans streptococcal adhesion and bracket bonding performance [43,44,45,46]. Finally, the sample-size strategy adopted for the primary endpoint is consistent with conventional statistical power analysis frameworks described by Faul et al. and Cohen [47,48].

The biological interpretation requires greater caution than in the initial submission. In the extract-based HGF-1 assay, cellular viability generally remained above the ISO 10993-5 screening threshold of 70%, but the UT-A group showed a statistically lower mean viability than untreated cells. More importantly, LDH increased in UT-F, UT-A, and P-A; IL-6 in UT-A; and IL-8 in UT-F and UT-A versus the untreated-cell reference. Thus, increases in LDH and inflammatory mediators cannot be dismissed solely because the coated-versus-polished contrasts were not statistically significant. The absence of a significant difference between coated and polished extracts is not proof of equivalence, particularly with six independent extracts per group and variable cytokine measurements. These short-term results do not establish in vivo mucosal safety or exclude effects under longer exposures [18].

The mechanical findings are relevant because even a surface-limited treatment should not compromise the function of the underlying appliance. Fresh MPC-PMMA and P-PMMA showed nearly identical flexural strength (84.9 ± 5.6 vs. 85.3 ± 5.4 MPa) and microhardness values (18.8 ± 1.4 vs. 19.1 ± 1.3). The same close agreement persisted after aging. Thus, the observed anti-biofilm effect was not accompanied by a meaningful loss of the measured bulk mechanical performance.

From a clinical perspective, this study does not demonstrate a reduction in caries, white spot lesions, or appliance-related mucosal disease because it remains an in vitro investigation. It does, however, provide a stronger translational screening framework than a single fresh-surface bacterial assay. A surface treatment that combines lower *S. mutans* colonization, demonstrable chemical persistence after aging, reduced protein adsorption, a measurable but non-equivalent short-term gingival cell response, and preserved mechanical performance merits subsequent evaluations using multispecies salivary biofilms, in situ exposure, and ultimately, controlled clinical studies.

Limitations include the monospecies 2 h/24 h microbiological model, the brief cell-extract exposure, the small number of independently prepared cell extracts, and the lack of a polymer-free sham-application control. T28, in the hydrolytic series, represents saliva immersion alone, whereas the—A groups were exposed to the complete aging sequence; their different endpoints and specimen hierarchies prohibit treating them as a single longitudinal series. The wettability/topography time course used repeated disk measurements; XPS used separate disks at each time, with technical readings not constituting additional independent replicates. Because disk-level XPS variability was unavailable, the reported XPS ± SD values are descriptive and do not support formal time point inference. Coating thickness was measured on separate reference silicon wafers rather than directly on PMMA disks. Matched-twin protein and microbial assays involved only six manufacturing batches and were sensitive to covariate adjustment and collinearity. Chemical surface persistence does not by itself resolve spatial coating defects or biofilm microstructure. This work did not assess polymicrobial plaque, caries outcomes, or clinical mucosal responses.

## 5. Conclusions

Within this in vitro study, photoreactive PMBPAz-MPC treatment of clinically polished orthodontic PMMA was associated with lower early *S. mutans* adhesion and 24 h biofilm indices than polishing alone. Surface chemical and wetting changes were smaller during saliva immersion alone (T0–T28) than after the full composite-aging challenge, following which the anti-adhesive difference remained measurable. These findings do not establish clinical plaque prevention or long-term biological safety.

## Figures and Tables

**Table 1:** Experimental groups and PMBPAz-MPC surface preparation.

Code	Base Material	Surface Condition	Preparation/Modification	Aging Condition	Role in Study
UT-F	Heat-polymerized PMMA	Untreated	Standard fabrication/finishing; no final clinical polishing; no coating	Fresh	Untreated surface control
UT-A	Heat-polymerized PMMA	Untreated	Same as UT-F	Composite aged	Untreated aging control
P-F	Heat-polymerized PMMA	Clinically polished	Standardized clinical polishing protocol	Fresh	Clinical surface control
P-A	Heat-polymerized PMMA	Clinically polished	Same as P-F	Composite aged	Polished aging control
MPC-F	Heat-polymerized PMMA	Polished + MPC coating	P-PMMA protocol + PMBPAz application + UV 254 nm for 2 min	Fresh	Experimental anti-fouling surface
MPC-A	Heat-polymerized PMMA	Polished + MPC coating	Same as MPC-F	Composite aged	Durability/efficacy group

**Table 2:** Simulated intraoral aging and coating-stability protocol.

Aging Module	Medium/Challenge	Temperature	Duration/Cycles	Renewal/Load/Frequency	Measurements
Hydrolytic stability series	Artificial saliva	37 °C	T0, T7, T14, T28	Standardized renewal schedule	MPC-PMMA: contact angle, SFE, XPS
Composite aging—hydrolytic phase	Artificial saliva	37 °C	28 days	Standardized renewal schedule	Pre/post surface characterization
Composite aging—thermocycling	Water baths	5 °C/55 °C	5000 cycles	Standardized dwell/transfer sequence	Coating/surface stability
Composite aging—toothbrushing	Standardized brushing simulation	Room temperature	Standardized cycle protocol	Standardized load and frequency	Coating/surface stability
Final aged endpoint	After all selected modules	—	—	—	CFU, CV, XTT, CLSM/SEM, protein adsorption, cell extracts, mechanical tests

**Table 6:** *S. mutans* outcomes by surface condition and aging status.

Group	2 h Adhesion (log10 CFU/cm^2^)	24 h Biomass (OD590)	24 h Metabolic Activity (OD450)	Biofilm Thickness (µm)	Biovolume (µm^3^/µm^2^)
UT-F	6.14 ± 0.29	1.18 ± 0.16	0.51 ± 0.08	42.6 ± 6.1	12.8 ± 2.1
UT-A	6.22 ± 0.31	1.26 ± 0.18	0.55 ± 0.09	45.3 ± 6.8	13.9 ± 2.4
P-F	5.71 ± 0.26	0.92 ± 0.14	0.39 ± 0.07	33.8 ± 5.2	9.6 ± 1.8
P-A	5.89 ± 0.28	1.03 ± 0.15	0.44 ± 0.08	37.1 ± 5.6	10.7 ± 1.9
MPC-F	4.98 ± 0.24	0.51 ± 0.11	0.19 ± 0.05	16.4 ± 3.7	4.1 ± 1.2
MPC-A	5.27 ± 0.27	0.64 ± 0.13	0.25 ± 0.06	20.2 ± 4.1	5.3 ± 1.4

## Data Availability

The original contributions presented in this study are included in the article. Further inquiries can be directed to the corresponding author.
